# Organisation, Financing, and Implementation of Preconception Care in Primary Care: Implications for Women’s Health and Well-Being

**DOI:** 10.3390/healthcare14172706

**Published:** 2026-08-25

**Authors:** Lora Draeva, Eleonora Hristova-Atanasova, Georgi Iskrov, Rumen Stefanov

**Affiliations:** Department of Social Medicine and Public Health, Faculty of Public Health, Medical University of Plovdiv, 4002 Plovdiv, Bulgaria; lora.draeva@mu-plovdiv.bg (L.D.); georgi.iskrov@mu-plovdiv.bg (G.I.);

**Keywords:** preconception care, women’s health, primary care, reproductive health, healthcare providers, financing, willingness to pay, implementation, narrative review

## Abstract

(1) Background: Preconception care (PCC) is an important component of preventive reproductive healthcare and public health because modifiable risk factors affecting maternal, neonatal, and longer-term health may be present before conception. Despite broad recognition of its potential value, substantial uncertainty remains regarding how PCC should be organised, financed, and implemented across different healthcare systems. (2) Methods: This structured narrative review synthesised evidence on healthcare- provider models, financing, affordability, willingness to pay (WTP), and barriers and facilitators affecting PCC implementation. A structured search of PubMed, Scopus, and Web of Science Core Collection covered publications from January 2010 to March 2026 and was updated on 14 July 2026. Original research studies, systematic and scoping reviews, narrative reviews, clinical guidelines, policy reports, and relevant methodological or health-economic publications were considered. A structured qualitative appraisal was used to interpret evidence according to study design or evidence type, directness to PCC, healthcare-system context, methodological limitations, and consistency across sources. A total of 61 publications were retained in the structured evidence synthesis. (3) Results: No single PCC delivery model was consistently supported as universally applicable. Across heterogeneous settings, the evidence more consistently supported broad principles including accessibility, continuity of care, multidisciplinary coordination, clear professional roles, financial accessibility, and integration with existing healthcare pathways. Implementation was constrained by insufficient training, consultation-time limitations, unclear professional responsibilities, fragmented service organisation, inadequate reimbursement, and socioeconomic inequalities. Evidence on WTP, specific financing arrangements, and digital delivery models was more limited and context-dependent. (4) Conclusions: The available evidence supports PCC as an important preventive reproductive-health strategy, but the optimal organisational and financing models remain uncertain and are likely to depend on healthcare-system context. Integrated and multidisciplinary approaches appear promising, although specific provider configurations, financing mechanisms, and digital interventions require further comparative and longitudinal evaluation. The author-developed hypothetical conceptual model proposed in this review should therefore be interpreted as a hypothesis-generating synthesis of the available evidence rather than as an empirically validated clinical pathway or causal model.

## 1. Introduction

Preconception care (PCC) is an important component of preventive reproductive healthcare and public health because many modifiable medical, behavioural, nutritional, psychosocial, genetic, and environmental risk factors affecting pregnancy and longer-term maternal and child health are present before conception [1,2,3,4,5,6]. PCC therefore provides an opportunity for early risk identification, counselling, health promotion, medication review, chronic disease optimisation, genetic assessment, and other preventive interventions before pregnancy occurs.

The public-health relevance of PCC is reinforced by the high prevalence of unplanned pregnancies and by the fact that important stages of embryonic development occur before many women initiate antenatal care [4,6,7,8,9,10]. This limits the opportunity to address preventable risks after conception and supports the integration of reproductive planning and preconception health into routine preventive healthcare.

Despite its potential benefits, PCC remains unevenly implemented across healthcare systems. Reported barriers include limited awareness, insufficient professional training, consultation-time constraints, fragmented service organisation, inadequate reimbursement, unequal access to preventive services, and uncertainty regarding professional responsibilities [1,3,7,8,11,12,13]. These challenges are particularly relevant because the feasibility of PCC depends not only on clinical recommendations but also on healthcare-system organisation, financing, workforce capacity, and accessibility.

There is also no universally accepted organisational model for PCC. General practitioners, obstetrician–gynaecologists, midwives, nurses, and other professionals may contribute complementary roles, while financing arrangements, affordability, willingness to pay (WTP), and implementation conditions influence whether services are accessible and sustainable [13,14,15]. Evidence concerning these dimensions remains heterogeneous across countries and healthcare systems.

This structured narrative review therefore synthesises evidence on healthcare-provider models, financing and affordability, WTP, and barriers and facilitators affecting PCC implementation, with particular attention to primary care, healthcare-system context, and implications for women’s health and well-being.

## 2. Materials and Methods

### 2.1. Study Design

This study was conducted as a structured narrative literature review of published evidence on the delivery, financing, and implementation of preconception care (PCC), with particular emphasis on provider preferences, willingness to pay (WTP), and implementation challenges in primary-care settings.

The methodological approach was informed by principles of narrative synthesis and by the Scale for the Assessment of Narrative Review Articles (SANRA) [16]. SANRA was used to inform the structure and reporting of the narrative review and was not applied as a risk-of-bias instrument for the included publications. The review was not designed as a systematic review, scoping review, or meta-analysis; consequently, no prospective protocol registration or quantitative synthesis was undertaken. Nevertheless, selected elements of systematic-review reporting were used as transparency benchmarks for documenting information sources, the search process, and publication selection, without implying formal compliance with the Preferred Reporting Items for Systematic Reviews and Meta-Analyses (PRISMA). This approach was considered appropriate because the evidence base comprised heterogeneous study designs, populations, healthcare systems, outcomes, and policy contexts. The purpose of the review was therefore to provide a structured and critical thematic synthesis across clinical, organisational, economic, and health-policy domains rather than to estimate pooled effects.

### 2.2. Search Strategy

A structured literature search was conducted in PubMed, Scopus, and Web of Science Core Collection. The principal database search covered publications from January 2010 to March 2026 and was updated on 14 July 2026 to identify additional relevant publications. Selected pre-2010 foundational methodological, economic, or policy publications were retained when directly relevant to WTP methodology, economic evaluation, or the conceptual framing of PCC. The database counts reported in the study-selection flow represent the final documented outputs following the July 2026 search update.

The search combined terms related to preconception care with terms addressing healthcare-provider organisation, primary care, multidisciplinary care, financing and affordability, willingness to pay, implementation barriers and facilitators, and digital or technological support. Free-text terms were combined using Boolean operators, and Medical Subject Headings (MeSH) were used in PubMed where applicable. Database-specific field syntax was applied according to the requirements of each database.

The original saved database search histories were not retained. During manuscript revision, the database-specific search formulations were retrospectively reconstructed from documented search terms and Boolean combinations used during the original search process and adapted to the syntax of each database. The reconstructed formulations, documented database outputs, search parameters, and search-update information are provided in Supplementary Table S1. Accordingly, the search formulations presented in Table S1 should be interpreted as transparent reconstructions of the original searches rather than as verbatim exports of the original database search histories.

In addition, reference lists of relevant publications were manually screened to identify potentially eligible publications not retrieved through the database searches.

### 2.3. Eligibility Criteria

Publications were considered eligible for inclusion in the structured evidence synthesis if they met the following criteria:

(1) original research studies, systematic reviews, scoping reviews, narrative reviews, clinical guidelines, policy or professional reports, and relevant methodological or health-economic publications;

(2) published between 2010 and 2026, with selected earlier foundational methodological, economic, or policy sources retained when directly relevant to WTP methodology, economic evaluation, or the conceptual framing of PCC;

(3) available in full text in English; 

(4) and addressed at least one predefined domain of the review, including healthcare-provider roles and organisation of PCC delivery, financing and reimbursement, affordability, service preferences, WTP, access and equity, or barriers and facilitators affecting implementation.

Publications directly addressing PCC were prioritised for inclusion. However, a limited number of publications from adjacent primary-care, reproductive-health, maternity, or prenatal-care settings were also retained when they provided relevant contextual evidence for predefined organisational or health-system domains for which PCC-specific evidence was limited, including interprofessional collaboration, financing, affordability, access, equity, or implementation. Such indirect evidence was identified explicitly during data extraction and was used only for contextual interpretation, not as evidence of PCC-specific clinical effectiveness.

Publications were excluded if they were available only as abstracts, editorials, commentaries, or letters without original data or substantial analytical content; focused exclusively on narrowly defined clinical subgroups without relevance to broader PCC organisation or implementation; addressed assisted reproductive technology without relevance to general PCC delivery; did not contribute to any predefined synthesis domain; or otherwise fell outside the stated eligibility framework.

Sources used only for general background, definitions, or methodological framing and not contributing to the structured evidence synthesis were cited in the manuscript where appropriate but were not counted among the 61 included publications.

### 2.4. Study Selection

All records identified through database searching and supplementary identification methods were considered against the predefined eligibility criteria. Records identified through reference-list screening and manual searches were merged with the database records before duplicate removal and were subsequently subjected to the same title/abstract and full-text eligibility procedures as the database-identified records. Screening was conducted in two stages: title and abstract screening followed by full-text assessment of potentially eligible publications. The initial title and abstract screening was performed by L.D. and subsequently reviewed by E.H.-A. Full-text eligibility assessment was undertaken by L.D. and E.H.-A. Disagreements or uncertainties concerning eligibility were resolved through discussion, with G.I. and R.S. consulted where necessary. Publications were retained when they met the eligibility criteria and contributed to at least one predefined synthesis domain, including the selectively retained indirect contextual evidence described above.

**Figure 1:**
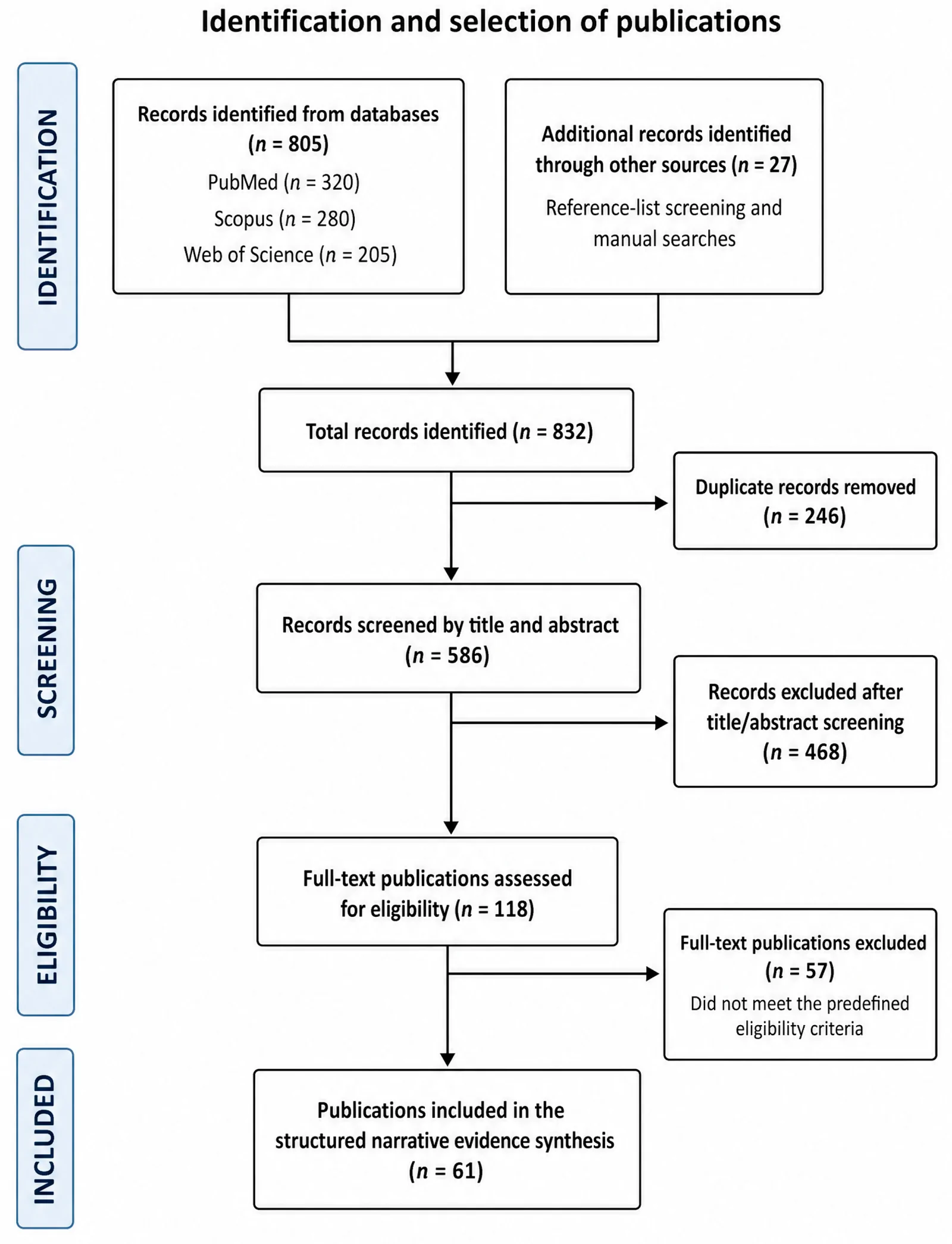
Simplified flow diagram of publication identification and selection for the structured narrative review. The diagram summarises database and supplementary identification, title/abstract screening, full-text eligibility assessment, and inclusion in the final evidence synthesis. It is provided to enhance methodological transparency and should not be interpreted as formal PRISMA reporting for a systematic review.

The final documented database outputs following the July 2026 search update comprised 805 records: 320 from PubMed, 280 from Scopus, and 205 from Web of Science Core Collection. An additional 27 publications were identified through reference-list screening and manual searches, yielding 832 records overall. After removal of 246 duplicate records, 586 records remained for screening. Of these, 468 were excluded following title and abstract screening, and 118 publications underwent full-text eligibility assessment. Following full-text assessment, 57 publications were excluded, and 61 publications were retained in the structured narrative evidence synthesis. The publication identification and selection process is summarised in Figure 1.

### 2.5. Data Extraction and Synthesis

A structured descriptive approach was used for data extraction and narrative synthesis. For each included publication, information relevant to the objectives of the review was extracted, including study design or evidence type, country or healthcare setting, population or data source, provider model, financing approach, affordability or WTP outcomes where applicable, implementation barriers or facilitators, principal findings, and reported methodological limitations.

The extracted evidence was organised into three predefined thematic domains: (1) healthcare-provider roles, organisation, and preferences in PCC delivery; (2) financing, affordability, service preferences, and WTP; and (3) barriers and facilitators affecting PCC implementation. Additional cross-cutting themes, including healthcare-system context, equity, multidisciplinary care, and digital or technological support, were incorporated where they contributed directly to interpretation of these domains.

Because the included evidence was heterogeneous in design, purpose, population, and outcome measurement, no formal quality-scoring system or quantitative risk-of-bias assessment was applied. Instead, a structured qualitative appraisal was used to guide interpretation. Each publication was considered in relation to its study design or evidence type, directness to PCC, population and healthcare-system context, reported methodological limitations, and consistency with other relevant sources.

Evidence from systematic or scoping reviews and directly relevant primary empirical studies was used preferentially when drawing broader conclusions, particularly when findings converged across multiple sources. Cross-sectional and qualitative studies were interpreted mainly as evidence of context-specific experiences, preferences, barriers, or implementation conditions. Clinical guidelines and professional recommendations were used to support clinical or consensus-based recommendations rather than comparative effectiveness claims. Narrative or conceptual reviews and policy or institutional documents were used primarily for conceptual, organisational, or policy contextualisation.

Indirect evidence from adjacent primary-care, reproductive-health, maternity, or prenatal-care settings was retained selectively only where it informed predefined organisational or health-system domains for which PCC-specific evidence was limited. Such evidence was identified explicitly in Table 1 and was not interpreted as evidence of PCC-specific clinical effectiveness.

The synthesis therefore distinguished findings that were relatively consistent across multiple sources from findings that were limited, heterogeneous, indirect, context-dependent, or emerging. The characteristics, principal findings, methodological limitations, and interpretative role of all publications included in the structured evidence synthesis are presented in Table 1.

### 2.6. Methodological Considerations and Search Limitations

Several methodological considerations should be noted when interpreting the review process. First, the review was designed as a structured narrative synthesis rather than as a systematic review; accordingly, no prospectively registered protocol, quantitative meta-analysis, or formal risk-of-bias assessment was undertaken. Transparency was instead strengthened through explicit eligibility criteria, documentation of publication selection, a simplified selection flow diagram, and structured qualitative appraisal of evidence type, directness, methodological limitations, and consistency across sources.

Second, the search was restricted to English-language full-text publications and to three bibliographic databases, supplemented by reference-list screening and manual searching. Relevant publications indexed elsewhere or published in other languages may therefore have been missed. In addition, the reconstructed database-specific search formulations presented in Supplementary Table S1 are based on documented search terms and Boolean combinations rather than retained verbatim database search histories.

Third, the heterogeneity of the included evidence in study design, population, healthcare-system context, and outcome definition limited direct comparison across publications and precluded quantitative synthesis. Publication bias and selective availability of evidence cannot be excluded, particularly where implementation experiences or unsuccessful programmes may be less likely to be reported.

## 3. Results

A total of 61 publications were retained in the structured evidence synthesis. The included evidence comprised primary empirical studies, systematic and scoping reviews, narrative and conceptual reviews, clinical guidelines, policy and professional documents, and health-economic publications. The characteristics of the included publications, including their geographical context, study design or evidence type, population or data source, principal findings, methodological limitations, and role in the evidence synthesis, are summarised in Table 1. Of the 61 publications, 55 directly addressed PCC or its organisational, clinical, implementation, policy, access, equity, or economic dimensions, whereas six publications provided indirect contextual evidence from adjacent healthcare settings.

### 3.1. Healthcare-Provider Models for PCC Delivery

The organisation of PCC delivery varies across healthcare systems, and the available evidence does not establish the superiority of a single provider model. The literature describes roles for general practitioners (GPs), obstetrician–gynaecologists (OB-GYNs), midwives, nurses, and multidisciplinary teams, with their relative contribution shaped by professional scope of practice, healthcare-system organisation, accessibility, and individual clinical needs [13,17,18,19,20,21].

#### 3.1.1. GPs

GPs are frequently identified as important potential providers of PCC within primary healthcare because routine general practice offers opportunities for longitudinal preventive care, chronic disease management, medication review, and identification of modifiable reproductive-health risks [22,23,24,25,26]. However, implementation in general practice remains inconsistent. Surveys and qualitative studies identify limited familiarity with PCC guidance, consultation-time constraints, insufficient training, limited resources, and competing preventive priorities as recurrent barriers [22,23,27]. Additional challenges include the infrequent attendance of women of reproductive age outside episodes of acute illness, financial constraints, and limited access to dedicated PCC services. Proposed strategies to improve PCC delivery include standardised checklists, written educational materials, clinical reminders, and informational posters that normalise discussions about reproductive intentions during routine consultations [22,27,28]. Among surveyed GPs, some conventional preventive topics such as smoking, alcohol use, vaccination, and medication review were addressed more consistently than overweight, mental health, or reproductive intentions [22].

#### 3.1.2. OB-GYNs

OB-GYNs play an important role in PCC because of their specialised expertise in reproductive medicine, fertility, pregnancy, and prevention of maternal and foetal complications. Their clinical expertise enables comprehensive assessment and optimisation of reproductive health before conception, particularly among women with pre-existing medical conditions or increased reproductive risk [29]. Specialist OB-GYN involvement is particularly relevant when preconception assessment requires management of complex reproductive or medical conditions, medication optimisation, evaluation of reproductive risk, or coordination of genetic counselling and carrier screening where indicated [29,30,31].

Their knowledge of medication safety and teratogenic risk also supports appropriate preconception medication review and therapeutic optimisation. Patient-preference studies indicate that specialist reproductive-health expertise may influence provider choice, although the magnitude of this preference varies across healthcare contexts [6,32]. Nevertheless, specialist-led PCC has limitations at the population level. Access to OB-GYN services outside pregnancy may depend on referral or existing reproductive concerns, reducing opportunities for timely universal prevention. Specialist involvement may be particularly relevant when clinical complexity exceeds the scope of routine primary-care PCC, although the evidence does not establish a universally superior specialist-led model [13,29,30,33].

#### 3.1.3. Midwives and Nurses

Midwives are increasingly recognised as important PCC providers because of their person-centred philosophy of care, emphasis on health promotion, and continuous engagement with women throughout the reproductive life course [17]. Their focus on partnership, education, and continuity creates favourable conditions for identifying preconception-health needs and delivering preventive interventions. Evidence also suggests a high level of professional readiness among midwives. Australian midwives reported substantial interest in providing preconception and interconception care, with 76% wishing to provide it more regularly and 87% considering it within midwifery scope of practice [34].

Potential contributions of midwives to PCC include health promotion, counselling, continuity, and person-centred support, although comparative evidence for midwife-led PCC remains limited [17,34]. However, their role varies considerably across healthcare systems and may be limited by regulatory, organisational, or funding constraints. Successful implementation therefore requires standardised education, continuing professional development (CPD), sustainable financing, and clear definition of professional responsibilities within multidisciplinary care pathways [34].

#### 3.1.4. Team-Based Models

A heterogeneous body of evidence identifies multidisciplinary coordination, role clarity, communication, and referral as recurrent organisational considerations in PCC, although comparative evidence for specific team configurations remains limited [13,21,35]. The most frequently described collaborative approaches involve GP–midwife and OB-GYN–midwife partnerships, although their structure and feasibility vary across healthcare systems. Collaborative GP–midwife arrangements are conceptually compatible with PCC because they may combine primary-care accessibility and longitudinal management with reproductive-health counselling. PCC-specific evidence supports the importance of role clarity, competence, communication, and referral pathways, but direct comparative evaluation of a defined GP–midwife PCC model remains limited [21,22,23,34]. Collaboration between OB-GYNs and midwives may provide complementary specialist and person-centred expertise. However, evidence specifically evaluating this configuration in PCC is sparse; maternity-care evidence concerning trust and communication should be interpreted only as indirect contextual evidence [29,30,31,36]. However, broader implementation may be constrained by lower accessibility to specialist services, dependence on referral pathways, and higher healthcare costs. Therefore, this model may be more appropriate for targeted management of women at increased reproductive risk than for universal population-based PCC delivery.

Across the available literature, several system-level conditions are repeatedly identified as potentially important for multidisciplinary PCC delivery, including clearly defined clinical pathways, explicit role allocation, postgraduate education and continuing professional development, interoperable electronic health records (EHRs) with automated reminders, digital risk-assessment tools, and sustainable reimbursement mechanisms [13,27,37,38]. However, the relative contribution of these components has rarely been evaluated comparatively. The evidence-supported contributions and contextual limitations of the principal PCC provider models, together with the authors’ interpretation of their potential roles within integrated PCC pathways, are summarised in Table 2.

#### 3.1.5. Women’s Preferences Regarding PCC Providers

Women’s preferences regarding PCC providers are shaped by accessibility, perceived professional competence, continuity of care, confidentiality, affordability, and the organisation of the healthcare system. Interest in PCC and preferences regarding its delivery have been reported across different settings, although preferred providers and service characteristics vary by healthcare-system context and population [6,32,41,42].

In one study, approximately 75% of women reported that they would be willing to use PCC services. In the same context, OB-GYNs were most frequently indicated as preferred providers (93%), followed by midwives (73%) and GPs (63%). As these categories were not necessarily mutually exclusive and reflect a specific healthcare context, the findings should be interpreted as evidence of perceived trust in reproductive health specialists rather than as universally generalisable provider preferences [6]. Similarly, an Australian discrete choice experiment (DCE) found a preference for face-to-face consultations, particularly when delivered by OB-GYNs, while provider choice was also influenced by consultation costs and financial incentives [32]. Across studies, accessibility, trust, confidentiality, and perceived competence emerge relatively consistently as determinants of provider preference, whereas preference for a specific professional group varies more substantially according to healthcare-system organisation and previous healthcare experience [41,42,43,44]. Women value easy access to care, respectful and non-judgemental communication, personalised counselling, and reliable information delivered within an established patient–provider relationship [42,43]. Reproductive experience has been associated with PCC knowledge and attitudes in observational evidence, although the magnitude and relevance of these associations vary across populations and outcome definitions [44,45]. Women have reported preferences for accessible, personalised and non-judgemental communication, with interest in multiple conventional and digital delivery formats [42,43,44]. The principal factors influencing women’s preferences for PCC providers, together with their variation across healthcare contexts and the relative consistency and directness of the supporting evidence, are synthesised in Table 3.

### 3.2. Financing, Affordability, Service Preferences, and WTP for PCC

#### 3.2.1. Current Financing Models

The financing of PCC varies substantially across healthcare systems, reflecting differences in health-policy priorities, healthcare organisation, insurance coverage, professional roles, and the extent to which preventive reproductive health is integrated into routine care. For comparative purposes, the financing arrangements identified in the included literature were grouped by the authors into four broad, non-mutually exclusive categories: publicly funded universal models, social or mandatory health-insurance models, mixed public–private systems, and fragmented or locally funded services. Across these arrangements, financial accessibility depends not only on nominal coverage but also on reimbursement mechanisms, service organisation, patient cost-sharing, and the availability of preventive healthcare resources [3,7,47,48]. Publicly funded healthcare systems may reduce direct financial barriers when PCC or related preventive reproductive-health services are incorporated into routine care, although implementation remains dependent on workforce capacity, professional roles, and service organisation. In the United Kingdom, national guidance provides a framework for preconception health, while implementation remains constrained by consultation time, competing clinical priorities, and limited public awareness [3,25]. European comparisons demonstrate substantial variation in the policy recognition, professional guidance, and organisation of PCC services across publicly financed and insurance-based systems [47,48]. These examples illustrate alternative organisational arrangements rather than evidence of superiority. In insurance-based primary-care systems, PCC responsibilities may be distributed across different healthcare professionals. In the Netherlands, PCC has been delivered through both general practice and midwifery, although dedicated PCC consultations remain inconsistently implemented [3,26]. In several other European settings, PCC remains more fragmented, with preventive reproductive-health activities depending on local programmes, professional practice patterns, or project-based initiatives rather than a uniform national pathway [3,47]. PCC-specific evidence on financing arrangements in Central and Eastern European settings remains limited, restricting direct cross-system comparison [3,23]. These findings indicate that financing should be interpreted together with healthcare-system capacity, service organisation, workforce availability, and referral infrastructure. In mixed public–private systems, insurance status and cost-sharing may contribute to inequalities in access to preconception and preventive reproductive healthcare. Evidence from the United States demonstrates socioeconomic and insurance-related disparities in preconception-health indicators and access to care, including among women with high-risk conditions [3,49,50]. Overall, the literature suggests that stronger financial protection and integration within existing healthcare structures may reduce access barriers, but available evidence does not establish the superiority of one financing model across healthcare systems. Financing arrangements are closely interrelated with insurance design, primary-care organisation, specialist access, workforce capacity, and national health policy.

#### 3.2.2. Economic Arguments for PCC

Direct economic evidence for PCC remains limited and heterogeneous; a systematic review identified only a small number of economic evaluations, with particularly sparse consideration of equity [15,51].

Earlier economic analyses have suggested potential cost savings from preconception interventions in defined high-risk populations, particularly women with pregestational diabetes. These estimates have been linked to the prevention of costly maternal and neonatal complications and reduced use of intensive healthcare resources. Earlier analyses suggest potential economic benefits for selected high-risk populations, particularly where preventable complications generate substantial downstream costs; however, these estimates are historical and context-dependent [37,51]. Potential economic consequences of PCC may extend beyond direct healthcare expenditure. Broader public-health analyses have linked investment in reproductive and maternal health with reductions in costs associated with unintended pregnancy, preventable healthcare utilisation, and adverse maternal or child outcomes, although these estimates are not specific to PCC alone [7]. Several modifiable risk factors relevant before conception—including smoking, obesity, poorly controlled chronic disease, harmful alcohol use, and selected psychosocial or mental-health risks—are associated with adverse pregnancy and longer-term health outcomes and may therefore contribute to downstream healthcare and societal costs [7,51]. The economic and financing dimensions of PCC are also closely related to equity. Socioeconomically disadvantaged populations may experience a higher burden of reproductive-health risks while simultaneously facing greater barriers to preventive care. Policy frameworks based on proportionate universalism therefore provide one possible approach in which universal access is combined with additional support for groups with greater medical or social vulnerability [7].

#### 3.2.3. Affordability, Service Preferences, and WTP for PCC

Evidence directly measuring WTP for PCC remains limited and heterogeneous. For this reason, the following synthesis distinguishes between direct WTP studies, discrete choice experiments assessing stated service preferences, studies of affordability and out-of-pocket costs, and indirect evidence concerning utilisation or service preferences. These outcomes provide related but conceptually distinct information and should not be interpreted interchangeably.

For methodological context, WTP and stated-preference approaches are briefly defined below using established health-economic methodological literature that was not part of the 61-publication structured evidence synthesis.

In health economics, WTP methods estimate the monetary value that respondents assign to a defined healthcare service or intervention. Contingent valuation methods typically ask respondents to state the maximum amount they would be willing to pay, whereas discrete choice experiments assess preferences between hypothetical service configurations that vary across attributes such as provider, cost, location, waiting time, or service content [52,68,69,70]. Interpretation of either approach requires clearly specified scenarios because stated values may change substantially according to the services included, the population studied, and the payment context [52]. Direct evidence on WTP for PCC itself is sparse. Indirect evidence from reproductive healthcare illustrates the importance of affordability in lower-resource settings. In Vietnam, 59.1% of respondents reported willingness to pay for reproductive-health services, but the average maximum WTP was substantially below the stated market price [53]. Because this study did not evaluate PCC specifically, its findings were used only to contextualise affordability and stated payment preferences rather than to estimate PCC-specific WTP. PCC-specific qualitative evidence from the Netherlands similarly indicates that direct patient charges may discourage use. Women, particularly those receiving social benefits, reported that they might seek free alternatives rather than pay direct consultation fees for PCC [54]. This finding supports the relevance of financial accessibility but represents stated intentions in a specific insurance context rather than observed utilisation behaviour. An Australian discrete choice experiment demonstrated that consultation cost and financial incentives influenced stated preferences for PCC service configurations. Respondents also showed preferences related to provider type and consultation modality, including face-to-face care [32]. These findings indicate that cost is an important attribute of PCC service design, but they should be interpreted as stated preferences rather than evidence of actual utilisation. WTP has also been examined for specific preconception interventions such as expanded carrier screening. In one mixed-methods study, higher stated WTP was associated with household economic circumstances and individual values, while reassurance regarding reproductive genetic risk was an important perceived benefit [52]. These findings relate to a specific genetic-screening context and should not be generalised to PCC services as a whole.

#### 3.2.4. Factors Associated with Affordability, Preferences, and WTP

The available evidence identifies several demographic, socioeconomic, reproductive, and service-related factors associated with PCC knowledge, utilisation, affordability, stated preferences, or WTP. Because these outcomes are not equivalent, the associations described below should be interpreted according to the type of outcome measured rather than as determinants of WTP alone. Age and educational attainment have been associated with PCC knowledge, attitudes, and utilisation across observational studies and evidence syntheses. Higher educational attainment is frequently associated with greater PCC knowledge or use, while age-related associations vary according to population and outcome definition [44,45]. These findings concern knowledge and utilisation more directly than WTP and should not be interpreted as evidence that education or age independently determines the amount individuals are willing to pay.

Economic capacity is relevant to affordability, but direct PCC-specific evidence remains limited. Indirect evidence from maternity and prenatal care demonstrates that substantial out-of-pocket expenditure may persist even where services are nominally free, creating financial burden for households [55,56]. These studies do not measure PCC utilisation or WTP and are therefore used only to illustrate the broader risk that patient cost-sharing may create access barriers within preventive reproductive healthcare. Reproductive history and pregnancy intentions have been associated with PCC utilisation and stated service preferences. Women with previous adverse pregnancy or infant outcomes may be more likely to use PCC before a subsequent pregnancy [57], while pregnancy intentions and service characteristics may influence stated preferences in discrete choice experiments [32]. These findings should not be interpreted as direct evidence that reproductive history determines WTP. In indirect reproductive-health WTP evidence, previous service use, occupation, perceived necessity of care, and other demographic or behavioural factors were associated with stated willingness to pay and maximum WTP [53]. Because this evidence was not PCC-specific, it informs only the broader interpretation of affordability and perceived value. Overall, the available evidence suggests that financial accessibility and service preferences are shaped by ability to pay, perceived need, healthcare experience, reproductive context, and service design. However, PCC utilisation, stated provider preferences, affordability, out-of-pocket expenditure, and maximum WTP represent distinct outcomes and should not be treated as interchangeable measures. Financing arrangements that rely heavily on direct patient payment may therefore raise equity concerns, particularly for socioeconomically vulnerable groups, although comparative evidence on the effect of specific PCC financing models remains limited.

### 3.3. Barriers and Facilitators to the Implementation of PCC

Barriers to PCC implementation operate across provider, organisational, health-system, technological, and patient levels. Across the included literature, barriers are documented more consistently than the comparative effectiveness of proposed facilitators, and many suggested solutions remain implementation-oriented rather than formally evaluated [13,23,27].

#### 3.3.1. Individual-Level Barriers

At the provider level, limited knowledge of PCC guidance, insufficient formal training, and variable confidence in initiating reproductive-health discussions are recurrent implementation barriers. In the Bulgarian GP survey included in this review, substantial gaps in PCC-specific training and service-management knowledge were reported, while studies from other settings similarly describe uncertainty regarding professional roles, guideline familiarity, and practical delivery [21,22,23,58]. Limited confidence and perceived opportunity may also constrain routine delivery. Because many women do not present specifically for PCC, providers may need to identify reproductive intentions opportunistically during routine consultations rather than relying on dedicated preconception visits [21,23]. Some healthcare professionals describe PCC as insufficiently embedded in routine practice, which may reduce the frequency with which reproductive intentions and preconception risks are discussed. These findings support the relevance of postgraduate education, continuing professional development, structured communication tools, and greater integration of PCC into routine primary-care encounters [21,23,58].

#### 3.3.2. Organisational Barriers

Organisational barriers include limited consultation time, high workload, insufficient resources, and fragmented service organisation. Consultation-time constraints are among the most consistently reported obstacles because comprehensive preconception assessment and counselling may be difficult to incorporate into routine visits focused on acute illness, chronic disease management, or competing preventive priorities [18,23,27]. Other reported organisational barriers include the absence of dedicated PCC encounters, insufficient staffing, fragmented multidisciplinary collaboration, inflexible appointment systems, transport or geographical barriers, and unclear allocation of responsibilities between GPs, midwives, OB-GYNs, and other professionals [18,23,27,58,59]. These barriers may be particularly relevant for socioeconomically disadvantaged, migrant, or medically vulnerable populations, although their relative importance varies substantially across healthcare settings [50,59]. Clear role allocation, structured referral pathways, and defined opportunities for opportunistic or dedicated PCC may therefore facilitate more consistent delivery [18,28,58].

#### 3.3.3. System-Level Barriers

At the health-system level, financing, governance, and the organisation of preventive care influence whether PCC can be incorporated into routine services. In settings where dedicated reimbursement or recognised payment mechanisms are absent, providers may have limited financial or organisational incentives to allocate additional time to comprehensive PCC. Qualitative and implementation evidence therefore identifies reimbursement and service recognition as potentially important system-level determinants of routine delivery [47,48,54,58]. Potential system-level facilitators described in the literature include public or insurance-based coverage, explicit reimbursement arrangements, incorporation of PCC into existing preventive programmes, and organisational recognition of PCC within routine service pathways. However, comparative evidence establishing which financing mechanism most effectively improves uptake or equity remains limited. Variation in national policy recognition and healthcare-system organisation also contributes to differences in PCC implementation across countries. Evidence from Central, Eastern, and South-Eastern European contexts remains comparatively limited, but available studies describe fragmented preventive pathways, workforce and training constraints, and uneven integration of PCC into routine primary care [3,23]. These findings should be interpreted as context-specific evidence rather than as a uniform description of the region.

#### 3.3.4. Digital Solutions and Technological Facilitators

Digital health technologies are increasingly being explored as potential facilitators of PCC implementation. The current evidence base primarily concerns feasibility, acceptability, user engagement, risk-assessment support, communication, and integration into clinical workflows rather than sustained behavioural or clinical effectiveness [17,60,61,62,63].

EHR-based data infrastructure may support monitoring and identification of preconception-health indicators, although its usefulness depends on data completeness, documentation practices, and local EHR structure [60]. Standardised screening tools and prompts represent a separate emerging implementation approach [38]. Mobile-health and telehealth approaches may also extend access to information, risk assessment, or counselling, particularly where conventional face-to-face services are difficult to access; however, evidence that they reduce financial, geographical, or organisational barriers in practice remains limited [17,61,62,63]. A virtual PCC programme, Frame Your Future, demonstrated high short-term engagement: 96% of participants created an account, 86% completed the digital risk assessment, 89% participated in virtual counselling, and 71% subsequently discussed preconception health with their physician. Nearly 80% reported greater confidence in discussing fertility-related issues, while approximately half stated that they would not otherwise have initiated such discussions [62]. These findings support feasibility and engagement but do not establish sustained behavioural or clinical effectiveness. Similarly, a Japanese pilot digital PCC programme reported high user-rated usefulness (92.8%) and improved understanding of preconception-health concepts (93.8%) [63]. Taken together, these studies suggest that digital interventions may complement conventional care by supporting engagement, information delivery, and selected aspects of communication. However, the available evidence remains based largely on pilot or feasibility studies and should not be interpreted as demonstrating sustained clinical effectiveness [17,61,62,63].

#### 3.3.5. Patient-Level Barriers

Patient-level barriers include limited awareness of PCC, low health literacy, financial constraints, unplanned pregnancy, and cultural or socioeconomic factors that may reduce perceived relevance, access, or engagement [41,46,64,65,66]. Evidence from multiple settings indicates that lack of awareness and limited understanding of preconception health are recurrent barriers, whereas financial, cultural, and socioeconomic barriers are more context-dependent [41,46,64,65,66].

Population-level facilitators proposed in the literature include community outreach, health education, normalisation of reproductive-intention discussions, and accessible communication through conventional or digital channels [11,17,65,66]. However, direct comparative evidence regarding the effectiveness of specific awareness or outreach strategies remains limited. The principal barriers to PCC implementation, together with potential facilitators and the consistency and context of the supporting evidence, are synthesised in Table 4.

### 3.4. Integrated PCC Framework

The evidence synthesised in this review suggests that PCC is best conceptualised as a coordinated preventive-care process rather than as a service assigned exclusively to a single professional group. Primary-care providers may support early identification of reproductive intentions, preventive risk assessment, chronic disease management, and initial counselling, while specialist OB-GYN, genetic, or other clinical expertise becomes increasingly relevant when reproductive, medical, genetic, or obstetric risk is identified. Midwives and other healthcare professionals may contribute complementary roles in health promotion, counselling, continuity, and patient-centred support, depending on professional scope of practice and healthcare-system organisation [21,23,29,30,34].

International recommendations identify medical and reproductive history, medication review, lifestyle and environmental exposures, genetic assessment where indicated, and optimisation of chronic disease or potentially teratogenic pharmacotherapy as relevant components of preconception assessment, although guideline content and evidentiary support vary across recommendations [8,29,30,39]. PCC-specific implementation studies identify role clarity, local collaboration, referral pathways, education, and organisational support as recurrent enabling conditions [21,23,27,34,58]. However, the optimal configuration of these components and professional roles has not been established through comparative PCC-specific studies.

Based on these converging but heterogeneous findings, we propose an author-developed hypothetical conceptual model in which provider organisation and multidisciplinary care interact with financing and affordability, implementation conditions, and digital support. Digital tools may support risk assessment, information delivery, communication, referral, and continuity of care, although current evidence predominantly concerns feasibility, acceptability, and engagement rather than sustained clinical effectiveness [17,60,61,62,63].

**Figure 2:**
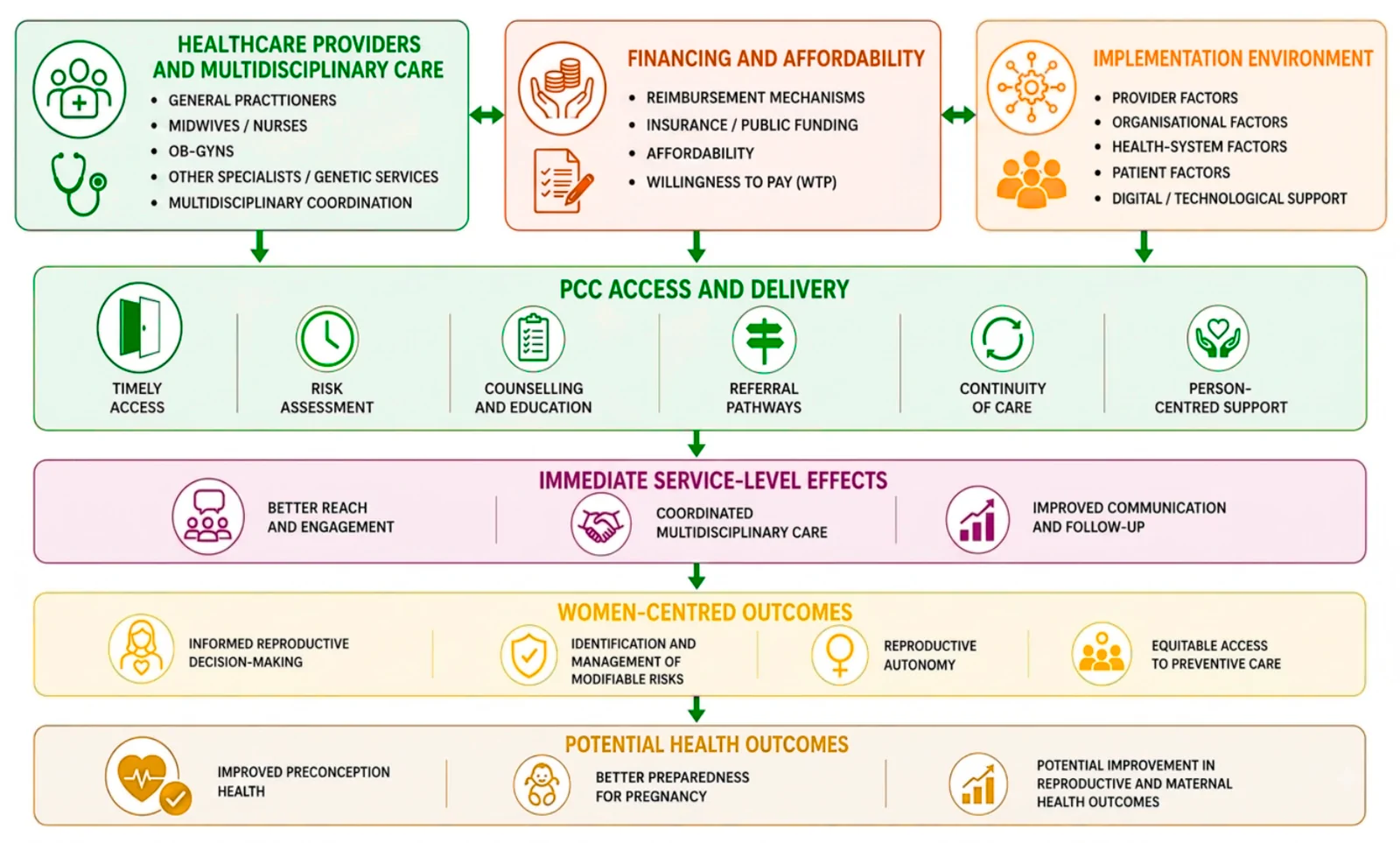
Author-developed hypothetical conceptual model of the organisation, financing, and implementation of comprehensive preconception care. Arrows indicate conceptual relationships proposed by the authors for hypothesis generation and should not be interpreted as empirically established causal pathways. The model was derived from the structured narrative synthesis and has not been externally or empirically validated.

The proposed conceptual model is intended to organise the available evidence and illustrate potential relationships between key implementation domains rather than to prescribe a universally applicable PCC pathway. The relationships shown in Figure 2 should therefore be interpreted as conceptual and hypothesis-generating rather than as empirically established causal pathways. The model has not been externally or empirically validated.

## 4. Discussion

### 4.1. Main Findings

The principal finding of this review is that the available evidence is more consistent in identifying broad conditions that may support PCC implementation than in demonstrating the superiority of specific organisational, financing, or delivery models. Across heterogeneous healthcare settings, accessibility, continuity, clear professional roles, coordinated referral, financial accessibility, and integration into routine care recur as important implementation themes [3,7,13,21,23,27,37]. By contrast, direct comparative evidence evaluating specific GP-led, midwife-led, specialist-led, multidisciplinary, reimbursement, or digital models remains limited.

The strength and directness of the evidence also differed substantially between review domains. Implementation barriers were supported by the most convergent body of evidence, particularly with respect to limited professional training, consultation-time constraints, uncertainty regarding professional responsibilities, fragmented service organisation, and insufficient or unclear reimbursement arrangements [13,18,21,23,27,58]. Evidence concerning provider roles and patient preferences was more context-dependent: women consistently valued professional competence, accessibility, continuity, trust, and respectful communication, but preference for a particular professional group varied across healthcare systems and service configurations [6,32,41,42,43,44].

The financing and economic evidence was less developed. Available studies support the relevance of affordability, patient cost-sharing, and financial protection, but the evidence does not establish a universally preferable financing model [7,47,48,51,54]. WTP evidence was particularly limited and heterogeneous, with substantial differences in study population, healthcare context, service definition, and stated-preference methodology [32,52,53,54]. Findings from indirect reproductive, maternity, or prenatal-care studies may help contextualise affordability and equity but should not be interpreted as PCC-specific economic effectiveness evidence [53,55,56].

Digital PCC represents an emerging rather than established implementation strategy. Current studies primarily demonstrate feasibility, acceptability, engagement, and support for risk assessment or communication, whereas evidence of sustained behavioural, reproductive, or clinical benefit remains limited [17,60,61,62,63]. Digital approaches should therefore be interpreted as potentially complementary components of PCC delivery rather than as validated substitutes for conventional clinical pathways.

Substantial heterogeneity across the evidence base was expected because studies differed in population, professional scope of practice, healthcare-system organisation, financing arrangements, intervention content, and outcome definition. Apparent inconsistencies between studies therefore often reflect differences in context and methodology rather than directly conflicting evidence. The synthesis consequently supports a set of adaptable organisational principles more strongly than a single transferable model of PCC delivery or financing.

Beyond its clinical and organisational dimensions, PCC may also contribute to women’s reproductive autonomy and well-being by supporting informed decision-making, timely access to counselling, and communication within respectful and non-judgemental healthcare relationships. These broader benefits should, however, be interpreted as part of the person-centred rationale for PCC rather than as outcomes that were consistently or quantitatively evaluated across the included literature.

### 4.2. Generalisability and Health-System Context

The applicability of the reviewed evidence is strongly influenced by healthcare-system context. Much of the available literature originates from high-income countries with established primary healthcare structures, relatively defined professional roles, and greater access to preventive, specialist, and digital services. In such settings, PCC can potentially be integrated into existing primary care, midwifery, reproductive-health, or insurance-based pathways. However, these organisational conditions cannot be assumed to exist in all healthcare systems.

In low- and middle-income countries (LMICs) and other resource-constrained settings, implementation may be shaped more strongly by workforce shortages, limited continuity of primary care, geographical barriers, lower availability of specialist services, out-of-pocket expenditure, competing maternal-health priorities, and differences in professional scope of practice. Available evidence from LMICs confirms important gaps in PCC knowledge, awareness, access, and implementation, but remains less extensive than the evidence from high-income settings [14,40,53,66]. Consequently, models relying on dedicated PCC consultations, specialist referral networks, extensive laboratory testing, or advanced digital infrastructure may require substantial adaptation before implementation in lower-resource environments.

Even among high-income countries, provider roles and financing arrangements differ substantially. Midwife-led models may be feasible where midwives have autonomous preventive and reproductive-health roles, whereas GP-led or shared-care models may be more appropriate in systems organised around strong primary care. Similarly, recommendations concerning reimbursement cannot be transferred directly between tax-funded, social-insurance, and mixed public–private systems. The findings of this review therefore support a set of transferable principles—early risk identification, continuity, multidisciplinary coordination, financial accessibility, and clear referral pathways—rather than a single universally applicable PCC delivery model. Local adaptation should reflect healthcare infrastructure, workforce capacity, financing arrangements, population needs, and the existing organisation of reproductive and primary healthcare. Accordingly, the limited representation of LMIC and resource-constrained settings should be considered not only a limitation of the current evidence base but also a priority for future comparative implementation research.

### 4.3. Implications for Practice

The high proportion of unplanned pregnancies globally underscores that PCC cannot be restricted to women who actively seek preconception counselling. A pragmatic implementation approach is to incorporate opportunities for PCC into routine healthcare contacts with people of reproductive age, including contraception, chronic disease, medication review, and preventive-care encounters [5,11,25,58].

From a clinical perspective, the findings support movement from opportunistic and provider-dependent counselling toward structured, risk-adapted PCC pathways with clearly defined responsibilities, referral mechanisms, and continuity across professional groups. Digital tools may support reach, communication, risk assessment, and continuity of care; however, current evidence primarily concerns feasibility and engagement rather than sustained clinical effectiveness [11,17,22,27,60]. PCC should also be conceptually and organisationally linked with antenatal care, while maintaining a clear distinction between evidence on preconception interventions and evidence derived from antenatal or maternity-care models. Findings from antenatal care research should not be interpreted as direct evidence for PCC effectiveness. Indirect interpregnancy and maternity-care evidence provides contextual support for continuity, communication, and coordinated referral across the reproductive-care continuum, but should not be interpreted as evidence of PCC-specific effectiveness [33,36,39,67].

### 4.4. Implications for Health Policy

Health-system financing and policy recognition may influence whether PCC becomes embedded in routine preventive care. In settings where dedicated reimbursement mechanisms, recognised service pathways, or explicit national guidance are absent or inconsistently implemented, providers may have fewer organisational and financial incentives to allocate time to comprehensive PCC. Sustainable implementation may therefore require explicit policy recognition of PCC within preventive and reproductive healthcare, although the optimal policy and reimbursement arrangements remain healthcare-system dependent [3,47,48,58]. National and health-system strategies have similarly emphasised the need to move PCC from professional recommendation toward routine, system-supported preventive care [7,12,47]. From an equity perspective, policy approaches based on proportionate universalism may be relevant, combining broad access to PCC with additional support for women and couples with greater medical, socioeconomic, or reproductive vulnerability. Public or insurance-based coverage and appropriately designed primary-care incentives may reduce financial barriers, but comparative evidence on the effects of specific financing mechanisms remains limited [3,7,58]. WTP should therefore be interpreted cautiously because stated willingness to pay may partly reflect ability to pay rather than underlying need or demand.

### 4.5. Future Research

Future research should move beyond documenting the importance of PCC and focus more directly on comparative implementation, financing, standardised evaluation, and long-term outcomes. First, comparative implementation studies are needed to determine how different PCC delivery models perform across healthcare systems with varying levels of primary-care capacity, specialist access, workforce availability, professional scope of practice, and insurance coverage. In particular, direct comparisons of GP-led, midwife-led, specialist-led, and multidisciplinary pathways remain scarce, limiting conclusions regarding the relative effectiveness, feasibility, and scalability of specific organisational models.

Second, the economic evidence remains underdeveloped. Further cost-effectiveness, cost–utility, budget-impact, affordability, willingness-to-pay, and equity-oriented analyses are required to determine how PCC can be financed sustainably and how different reimbursement arrangements influence access and uptake. Such evaluations should distinguish between expressed willingness to pay and actual ability to pay, particularly among socioeconomically vulnerable populations.

Third, substantially more evidence is needed from low- and middle-income countries and other resource-constrained healthcare systems. Future studies should examine how workforce shortages, limited primary-care infrastructure, out-of-pocket expenditure, geographical barriers, digital access, and competing maternal-health priorities influence the feasibility and adaptation of PCC models in these settings.

Fourth, the field would benefit from greater standardisation of implementation outcomes. Studies currently use heterogeneous definitions and indicators of PCC delivery, making comparison across settings difficult. Consensus-based indicators are needed to evaluate domains such as population reach, identification of reproductive intentions, uptake of risk assessment and counselling, referral completion, continuity of care, provider participation, financial accessibility, equity, digital engagement, and integration into routine healthcare. Development of a core set of PCC implementation indicators would facilitate benchmarking and comparative evaluation across healthcare systems.

Finally, longitudinal and prospective studies are required to determine whether structured PCC programmes produce sustained improvements beyond short-term knowledge, acceptability, or engagement. Relevant outcomes should include behavioural risk modification, pregnancy planning, uptake of genetic counselling and preventive interventions, reproductive and maternal health outcomes, patient-reported outcomes, equity of access, and longer-term health and economic consequences. These research priorities are essential for distinguishing promising organisational approaches from models that demonstrate durable effectiveness and scalability in routine practice.

### 4.6. Strengths and Limitations

A strength of this review is the integration of clinical, organisational, economic, implementation, and health-policy evidence within a common analytical framework, together with explicit consideration of study design, directness to PCC, healthcare-system context, and methodological limitations. The search was updated to July 2026, and publication selection, evidence characteristics, and the interpretative role of direct and indirect evidence were documented transparently.

Several limitations should also be considered. First, the review was designed as a structured narrative synthesis and did not include a prospectively registered protocol, formal risk-of-bias assessment, or quantitative meta-analysis. Second, the search was restricted to three bibliographic databases and English-language full-text publications. Relevant evidence indexed elsewhere or published in other languages may therefore have been missed. Third, the original database search histories were not retained; the database-specific formulations reported in Supplementary Table S1 were retrospectively reconstructed during manuscript revision from documented search terms and Boolean combinations. Fourth, substantial heterogeneity in study design, population, healthcare-system organisation, financing arrangements, intervention content, and outcome definition limited direct comparison across publications. Finally, the predominance of evidence from high-income settings and the selective use of indirect contextual evidence limit the transferability of some findings to resource-constrained healthcare systems.

## 5. Conclusions

The available evidence supports PCC as an important component of preventive reproductive healthcare and women’s health. Across heterogeneous healthcare settings, relatively consistent findings support broad principles including early identification of reproductive and medical risks, continuity of care, multidisciplinary coordination, financial accessibility, and clear referral pathways.

Specific organisational approaches, including GP-led, midwife-led, specialist-led, multidisciplinary, digitally supported, and alternative financing models, appear potentially valuable but remain incompletely evaluated comparatively. Their effectiveness, feasibility, scalability, and equity implications are likely to depend on healthcare-system organisation, workforce capacity, professional scope of practice, financing arrangements, digital infrastructure, and population needs.

The author-developed hypothetical conceptual model proposed in this review is intended to organise the available evidence and identify implementation priorities. It is hypothesis-generating, has not been externally or empirically validated, and should not be interpreted as a prescriptive clinical pathway or as evidence of established causal relationships.

## Figures and Tables

**Table 1:** Characteristics, principal findings, limitations, and role of publications included in the structured narrative evidence synthesis (*n* = 61).

First Author, Year	Country/Region	Study Design/Evidence Type	Population/Data Source and Sample Size	PCC Domain/Main focus	Principal Findings/Contribution	Key Limitations	Role in the Evidence Synthesis
Hristova-Atanasova et al., 2025 [3]	Global/Bulgaria	Narrative review	Global evidence with specific consideration of the Bulgarian healthcare context; N/A	Healthcare-system organisation; genetic screening; implementation	Synthesises global evidence on PCC-linked genetic screening and discusses implementation perspectives relevant to Bulgaria.	English-language evidence; limited direct Bulgarian evidence; reliance on heterogeneous international studies.	Narrative/conceptual PCC synthesis; used for comparative health-system and implementation contextualisation.
World Health Organization, 2013 [5]	Global	Policy brief	Global PCC policy and intervention framework; N/A	Policy; implementation; service delivery	Provides a global framework for PCC and describes promotive, preventive and curative interventions across the life course.	Policy/consensus source; not a comparative empirical evaluation.	Policy/contextual PCC evidence; used to inform global implementation principles.
Goossens et al., 2016 [6]	Belgium	Cross-sectional survey	Women of reproductive age with a desire for future children; *n* = 242	Patient needs; provider preferences; uptake	Approximately 75% expressed willingness to use PCC; OB-GYNs were most frequently selected as preferred providers, followed by midwives and GPs.	Self-reported preferences; single-country setting; provider categories were not necessarily mutually exclusive.	Direct empirical PCC evidence; patient needs and provider preferences.
Public Health England, 2018 [7]	United Kingdom	Policy report	National PCC policy evidence base; N/A	Financing; economic rationale; implementation	Develops the public-health and economic case for PCC and emphasises coordinated pathways across reproductive and maternal healthcare.	Policy/contextual evidence; limited primary empirical data.	Policy/contextual PCC evidence; financing and national implementation context.
Dorney et al., 2022 [8]	International/multi-country	Systematic review of clinical guidelines	11 clinical PCC guidelines	Guideline quality; PCC recommendations	Identified substantial variation in guideline quality and in the strength of evidence underpinning different PCC recommendations.	Heterogeneity in guideline scope, methodology and supporting evidence.	Synthesis-level PCC guideline evidence; used to assess consistency and strength of clinical recommendations.
Hall et al., 2023 [11]	United Kingdom	Evidence-informed conceptual/health-policy model	Community reproductive-health evidence and service-model literature; N/A	Integrated contraception and PCC; life-course delivery	Proposes an integrated community-based approach linking contraception and PCC to reproductive-health needs across the life course.	Conceptual/service-model evidence; no direct comparative effectiveness assessment.	Conceptual/health-policy synthesis; organisational integration and life-course PCC delivery.
Verbiest et al., 2016 [12]	United States	Narrative/national strategy paper	US Preconception Health and Health Care Initiative; N/A	Policy; implementation strategy	Reviews progress in US preconception health and identifies strategies for advancing PCC implementation and system change.	Narrative and country-specific; no comparative effectiveness analysis.	Narrative/policy PCC evidence; national implementation and system-change context.
Goossens et al., 2018 [13]	Multi-country	Systematic review	Healthcare providers; heterogeneous included studies	Provider barriers and facilitators	Provider-related literature identified recurrent barriers at patient, professional, organisational and societal levels, with barriers generally outweighing facilitators.	Heterogeneous study designs, professions and healthcare settings.	Synthesis-level PCC evidence; major source for the consistency of provider and implementation barriers.
Doe et al., 2026 [14]	Africa/multi-country	Systematic review	27 studies involving women of reproductive age	Awareness; knowledge; access; patient/system barriers	PCC awareness and knowledge varied widely across African settings; education, socioeconomic circumstances, healthcare access and counselling were important determinants.	Substantial methodological and country-level heterogeneity; predominantly observational evidence.	Synthesis-level PCC evidence; African/LMIC patient-barrier and generalisability evidence.
Carrandi et al., 2022 [15]	International	Systematic review of economic evaluations	8 economic evaluations	Health economics; equity; financing	Found a limited PCC economic evidence base and particularly sparse consideration of equity in economic evaluations.	Small and heterogeneous evidence base; limited equity analysis.	Synthesis-level PCC economic evidence; informs economic evidence and research gaps.
Nypaver & Yeager, 2024 [17]	International/predominantly US clinical context	Narrative review	General PCC literature; N/A	Innovative delivery; digital PCC	Describes innovations including apps, EHR reminders, group approaches and other strategies as promising methods for expanding PCC delivery.	Narrative evidence; limited comparative and long-term effectiveness evaluation.	Narrative PCC synthesis; emerging digital and service-delivery approaches.
M’hamdi et al., 2017 [18]	Netherlands	Qualitative study	Healthcare providers; *n* = 20	Provider and organisational barriers	Providers reported organisational and role-related barriers, including ambiguity concerning professional responsibility for PCC delivery.	Small purposive sample; single healthcare-system context.	Direct empirical PCC evidence; provider and organisational barriers.
Caut et al., 2025 [19]	Multi-country	Systematic review	Healthcare professionals; multiple included studies	Professional beliefs, attitudes and implementation barriers	Consultation time and other recurrent implementation barriers persisted across professions and settings, while many professionals considered PCC within their role.	Heterogeneous study designs, professional groups and healthcare systems.	Synthesis-level PCC evidence; provider attitudes and consistency of barriers.
Bortolus et al., 2017 [20]	Italy	Qualitative focus-group study	14 women + 12 healthcare professionals	Awareness; provider/patient barriers	Found low awareness of preconception health among both women and professionals, together with practical and communication-related barriers.	Small purposive qualitative sample; single-country setting.	Direct empirical PCC evidence; patient and provider barriers.
Maas et al., 2022 [21]	Netherlands	Mixed-methods study	Multidisciplinary healthcare providers; *n* = 250	Community implementation; professional roles	Providers emphasised role clarity, collaboration, local organisation and educational support as important for improving PCC implementation.	Local implementation setting; self-reported and consensus-oriented components.	Direct empirical PCC evidence; community-level implementation and multidisciplinary organisation.
Kizirian et al., 2019 [22]	Australia	Cross-sectional survey	General practitioners; *n* = 110	GP knowledge; provider role; implementation	Only approximately half of surveyed GPs were familiar with contemporary PCC guidance; limited time and resources were recurrent barriers.	Self-reported data; single-country professional sample.	Direct empirical PCC evidence; GP knowledge, readiness and barriers.
Hristova-Atanasova et al., 2024 [23]	Bulgaria	Cross-sectional survey	General practitioners; *n* = 116 of 420 invited; response rate 27.6%	Provider roles; PCC service management	Bulgarian GPs demonstrated partial familiarity with FP/PCC service management and reported gaps in training and organisational support.	Self-report; relatively low response rate; single-country setting.	Direct empirical PCC evidence; Bulgarian/CEE primary-care implementation context.
Wilkes, 2016 [24]	United States	Professional policy/position report	AAFP position statement; N/A	Primary-care provider role	Summarises the AAFP position supporting family physicians’ role in PCC delivery.	Professional-position evidence; limited direct comparative evaluation.	Policy/position evidence; contextualises the family-physician/primary-care role.
Stephenson et al., 2021 [25]	United Kingdom	Clinical/narrative review	General-practice population; N/A	Primary-care integration	Argues for stronger incorporation of preconception-health promotion into routine primary care and describes existing implementation as inconsistent.	Narrative/clinical review; UK-specific context; no comparative effectiveness evaluation.	Clinical/narrative PCC evidence; primary-care integration context.
Van Voorst et al., 2016 [26]	Netherlands	Cross-sectional survey	Primary-care providers; 449 GPs + 250 midwives	Provider roles; current PCC practice	Fewer than half reported having a PCC protocol; risk-factor identification and delivery practices varied substantially between providers.	Self-reported practice; single-country setting; possible non-response bias.	Direct empirical PCC evidence; primary-care organisation and implementation.
Mazza et al., 2013 [27]	Australia	Qualitative study	General practitioners; *n* = 22	Guideline implementation; provider barriers	GPs identified limited consultation time, insufficient training and competing preventive priorities as major barriers to PCC implementation.	Small qualitative sample; country-specific practice context.	Direct empirical PCC evidence; GP guideline-implementation barriers.
Nacev et al., 2022 [28]	United States	Qualitative study	Healthcare providers serving reproductive-age populations; *n* = 20	Provider behaviour; implementation	PCC delivery behaviour was influenced by guideline knowledge, workflow, training, professional role and perceived patient receptivity.	Small qualitative sample; US outpatient context.	Direct empirical PCC evidence; determinants of provider PCC delivery behaviour.
ACOG, 2019 [29]	United States	Clinical guideline/committee opinion	Expert/committee consensus; N/A	Provider roles; prepregnancy counselling	Provides recommendations concerning the content and delivery of prepregnancy counselling and the role of obstetric-gynaecological care.	Guideline/consensus evidence rather than primary empirical evidence.	Guideline/consensus evidence; informs clinical PCC components and specialist roles.
Giouleka et al., 2025 [30]	Multi-country guideline context	Comparative guideline review	Selected major national/international PCC guidelines; N/A	Guideline comparison; service organisation	Demonstrates variation in recommended PCC content, risk assessment and delivery approaches across major guideline sources.	Selected guideline comparison; not a systematic comparative effectiveness evaluation.	Comparative guideline synthesis; demonstrates variation in PCC recommendations and organisation.
ACOG Committee Opinion No. 690, 2017 [31]	United States	Clinical guideline/committee opinion	Expert/committee consensus; N/A	Genetic screening; carrier screening	Provides consensus recommendations concerning expanded carrier screening within contemporary reproductive and preconception care.	Guideline/consensus source; not primary empirical evidence.	Guideline/consensus evidence; supports genetic-screening components of PCC.
Haas et al., 2026 [32]	Australia	Discrete choice experiment	Australian adults recruited through an online panel; *n* = 485	Provider preferences; service design; WTP	Respondents preferred face-to-face PCC, particularly when delivered by specialist obstetricians; lower out-of-pocket costs and financial incentives also influenced stated choices.	Hypothetical/stated choices rather than observed service utilisation.	Direct empirical PCC evidence; stated preference/DCE evidence on provider choice and financing.
Dean et al., 2014 [33]	Global	Narrative/commentary	Global PCC evidence; N/A	Implementation; continuum of care	Identifies PCC as an insufficiently implemented component of the maternal–child health continuum and discusses strategies for closing the implementation gap.	Narrative synthesis; no formal comparative appraisal.	Narrative/global PCC synthesis; implementation framing.
Bradfield et al., 2023 [34]	Australia	Cross-sectional survey	Midwives; *n* = 338	Midwifery role; knowledge; readiness	Most respondents considered PCC within the scope of midwifery, and many wished to provide it more frequently; experience was associated with self-reported competence.	Self-reported data; single-country sample.	Direct empirical PCC evidence; midwifery role, readiness and training needs.
Bates et al., 2025 [35]	Global	Scoping review	Multidisciplinary primary-care teams; 27 included studies	Team-based service organisation	Found mixed evidence concerning multidisciplinary primary-care teams, with substantial variation in team composition and implementation across settings.	Not PCC-specific; heterogeneous designs and team models.	Indirect synthesis-level evidence; used only to contextualise multidisciplinary primary-care organisation, not PCC-specific effectiveness.
El Hussein et al., 2024 [36]	Canada	Concurrent mixed-methods study	Midwives; survey and semi-structured interviews (*n* = 18 survey; *n* = 13 interviews)	OB-GYN–midwife collaboration	Midwives reported generally positive but variable interprofessional collaboration with obstetricians, strongly influenced by trust and communication.	Small sample; maternity rather than PCC-specific setting; single-country context.	Indirect empirical evidence from maternity care; used only to contextualise OB-GYN–midwife collaboration and interprofessional communication.
Mason et al., 2014 [37]	Global/multi-country	Narrative/commentary	Global PCC evidence; N/A	Implementation; economic rationale	Discusses moving PCC from recognition toward implementation and highlights potential economic benefits, particularly for defined high-risk groups.	Narrative source; economic estimates largely derived from earlier and context-specific analyses.	Narrative/global PCC synthesis; implementation and economic contextualisation.
Ren et al., 2023 [38]	Predominantly United States evidence base	Systematic review	Identified 53 eligible studies representing 22 screening tools or standardised approaches; 10 had evidence from randomised clinical trials.	Preconception/interconception screening tools	Identified substantial heterogeneity in screening-tool content, implementation, and validation; 10 of the 22 tools or standardised approaches had evidence from randomised clinical trials.	Heterogeneous tools, outcomes and validation approaches; limited long-term effectiveness evidence.	Synthesis-level PCC evidence; screening-tool implementation and evidence heterogeneity.
Obstetric Care Consensus No. 8, 2019 [39]	United States	Clinical guideline/consensus	Expert/committee consensus; N/A	Interpregnancy care; continuity	Provides recommendations for interpregnancy health management that complement prepregnancy counselling and risk optimisation.	Consensus-based interpregnancy guidance with direct relevance to the preconception–interpregnancy continuum; not comparative empirical evidence.	Guideline/consensus evidence used to inform continuity of care and risk optimisation across the preconception–interpregnancy continuum; not used as comparative effectiveness evidence.
Aynalem et al., 2025 [40]	LMICs/multi-country	Scoping review	Reproductive-age individuals, healthcare workers and stakeholders; 62 included studies	Knowledge, attitudes and practices; LMIC barriers	Demonstrates substantial variation in PCC KAP and identifies socioeconomic, cultural, professional and health-system barriers across LMICs.	Heterogeneous countries, populations and study designs; limited direct comparability.	Synthesis-level PCC evidence; principal source for LMIC implementation and generalisability.
Poels et al., 2016 [41]	Multi-country/predominantly high-income settings	Systematic review	Women; multiple included studies	Barriers and facilitators to PCC uptake	Identifies limited awareness, access barriers and insufficient provider-initiated discussion among recurrent reasons for low PCC utilisation.	Heterogeneous study designs, populations and settings.	Synthesis-level PCC evidence; patient-level barriers and facilitators.
Daly et al., 2024 [42]	England, United Kingdom	Qualitative study	Women recruited from a preceding survey; 20 interviews	Patient preferences; content and delivery of PCC interventions	Women favoured accessible, personalised and non-judgemental approaches to preconception-health communication and intervention delivery.	Small qualitative sample; regional context; limited transferability.	Direct empirical PCC evidence; patient preferences and acceptable delivery approaches.
Sardasht et al., 2022 [43]	Iran	Qualitative study	13 reproductive-age women + 12 midwives	PCC quality; patient/provider preferences	Trust, confidentiality and personalised counselling emerged as important features of acceptable, high-quality PCC.	Small purposive sample; culturally and nationally specific findings.	Direct empirical PCC evidence; trust, privacy and perceived quality.
Daly et al., 2022 [44]	England	Cross-sectional survey	Women; *n* = 835	Knowledge; attitudes; delivery preferences	PCC knowledge and attitudes varied by sociodemographic characteristics; women expressed interest in receiving information through multiple delivery formats.	Self-reported data; single-country setting.	Direct empirical PCC evidence; patient KAP and delivery preferences.
Hidayah et al., 2025 [45]	Multi-country	Systematic review/meta-analysis	Women of reproductive age represented in 13 observational studies	Determinants of PCC KAP	Higher education, older age, previous PCC training or counselling, reproductive experience, and selected indicators of healthcare access were associated with greater PCC knowledge, more favourable attitudes, or better reported practices, although substantial heterogeneity was present for several associations.	Heterogeneity in designs, settings and outcome definitions.	Synthesis-level/meta-analytic PCC evidence; determinants of patient KAP.
Craig et al., 2025 [46]	Multi-country	Cross-sectional survey	Adults from five economically diverse countries; *n* = 5000	Knowledge; attitudes; behavioural intentions	Preconception-health knowledge, attitudes, behavioural intentions, and preferred sources of information varied significantly across five economically diverse countries, supporting strong context dependence of population-level PCC preferences.	Self-reported data; five-country sample cannot represent all global healthcare contexts.	Direct multi-country PCC evidence; cross-system variation in KAP and behavioural intentions.
Shawe et al., 2015 [47]	Belgium (Flanders), Denmark, Italy, Netherlands, Sweden and United Kingdom	Comparative descriptive policy and service review	PCC policies, guidelines and services in six European countries; N/A	Healthcare-system organisation; policy; financing	Identified substantial cross-country variation in PCC policies, professional recommendations, service organisation, and the availability and content of guidance for healthy populations.	Descriptive policy comparison; not an effectiveness evaluation.	Comparative PCC health-system evidence; key source for generalisability and system variation.
Berglund & Lindmark, 2016 [48]	Sweden	Narrative/strategy paper	Swedish PCC/PHC context; N/A	Healthcare-system organisation; financing	Describes the Swedish approach to preconception health, including an accessible role for midwifery and preventive reproductive-health services.	Contextual single-country strategy evidence.	Policy/strategy evidence; Scandinavian health-system context.
Rokicki & McConnell, 2024 [49]	United States	Secondary-data analysis	Women; *n* = 123,697	Equity; preconception-health disparities	Demonstrated persistent racial and socioeconomic differences in preconception-health risks that were not uniformly attenuated by higher income.	Observational secondary data; residual confounding; US-specific social and healthcare context.	Direct preconception-health equity evidence; access and equity contextualisation.
Easter et al., 2017 [50]	United States	Retrospective cohort study	Pregnant women with pregestational type 1 or type 2 diabetes receiving multidisciplinary endocrinology and obstetric care; *n* = 197	Access; insurance disparities; high-risk PCC	Publicly insured women experienced important gaps in preconception and pregnancy-related care compared with more advantaged insurance contexts.	High-risk clinical population; observational design; insurance-specific US setting.	Direct PCC evidence in a high-risk population; insurance/access disparities.
Grosse et al., 2006 [51]	United States/economic-policy context	Economic/methodological analysis	Foundational PCC economic literature; N/A	Economic evaluation; business case for PCC	Examines the evidence and methodology underlying the economic case for PCC, with particularly strong historical rationale for selected high-risk populations.	Older evidence; economic estimates depend on assumptions, population and payer perspective.	Foundational PCC economic evidence; retained under the predefined pre-2010 economic exception.
Clarke et al., 2018 [52]	United States	Mixed-methods study	Women/couples undergoing preconception carrier screening; *n* = 277 WTP survey; *n* = 58 interview subsample	WTP; genetic screening	Household economic circumstances and individual values were associated with WTP for expanded carrier screening; reassurance was an important perceived benefit.	Stated WTP; selected screening population; context-specific preferences.	Direct empirical PCC evidence; WTP for preconception genetic screening.
Nguyen et al., 2023 [53]	Vietnam	Cross-sectional survey	Adults in an urban–rural transition setting; *n* = 883	WTP; affordability; reproductive-health services	59.1% reported willingness to pay for reproductive-health services; average maximum WTP was substantially below the stated market price.	Not PCC-specific; stated rather than observed payment behaviour; single-country context.	Indirect economic evidence from reproductive healthcare; used only to contextualise affordability and WTP in an LMIC setting, not as PCC-specific WTP evidence.
Van Voorst et al., 2017 [54]	Netherlands	Qualitative study	Consumers/women; *n* = 39	Service design; affordability; provider preferences	Women, particularly those receiving social benefits, indicated that direct consultation fees could lead them to seek free alternatives rather than paid PCC services.	Small purposive sample; stated intentions; context-specific financing environment.	Direct empirical PCC evidence; affordability and patient preferences for service design.
Acharya, 2016 [55]	Nepal	Hospital-based cross-sectional study	Postpartum women and families using nominally free maternity services; *n* = 384	Financing; out-of-pocket costs; equity	Demonstrated that nominally free maternity services could still generate substantial direct and indirect household expenditure.	Maternity rather than PCC-specific population; hospital-based single-country study; no PCC outcomes.	Indirect financing/equity evidence from maternity care; used only to illustrate residual out-of-pocket burden despite nominally free healthcare.
Gunarathne et al., 2021 [56]	Sri Lanka	Cross-sectional study	Women attending their first prenatal clinic visit; *n* = 1389	Financing; out-of-pocket expenditure	Despite free maternal healthcare, women incurred measurable out-of-pocket expenditure at the first prenatal visit.	Prenatal rather than PCC-specific population; single-country setting.	Indirect financing/equity evidence from prenatal care; used only to contextualise residual patient costs within publicly funded healthcare.
Batra et al., 2016 [57]	United States	Secondary survey-data analysis	Women with a previous adverse infant outcome; sample defined by LAMB survey dataset	PCC uptake; reproductive history	Previous adverse infant outcomes were associated with greater use of PCC before a subsequent pregnancy.	Self-reported survey data; local US population; observational association.	Direct empirical PCC evidence; predictors of PCC utilisation.
Poels et al., 2017 [58]	Netherlands	Qualitative Nominal Group Technique study	Community healthcare providers; *n* = 30	Provider roles; reimbursement; implementation	Lack of reimbursement and unclear allocation of professional responsibility were identified as major barriers to routine PCC delivery.	Small local professional sample; context-specific service organisation.	Direct empirical PCC evidence; organisational roles and reimbursement barriers.
McGranahan et al., 2024 [59]	England	National secondary cross-sectional analysis	Women with an antenatal booking appointment recorded in the NHS Maternity Services Data Set, England; *n* = 652,880 (migration-category data available for *n* = 432,022)	Equity; migrant women; preconception health	Identified inequalities in important preconception-health indicators among migrant women, particularly those in vulnerable circumstances.	Reliance on routine maternity data; incomplete migration information; indicators are not equivalent to direct PCC utilisation.	Direct preconception-health equity evidence; vulnerable-population and access context.
Williams et al., 2022 [60]	United States	Retrospective EHR data analysis	Patients receiving prenatal or primary care in two North Carolina health systems; *n* = 15,384 at Site 1 and *n* = 6983 at Site 2 (total *n* = 22,367)	Digital/data infrastructure; PCC monitoring	Demonstrated substantial variation in documentation of preconception-wellness indicators between health systems, complicating consistent EHR-based measurement.	Dependent on routine documentation and EHR structure; measures monitoring feasibility rather than PCC effectiveness.	Direct PCC implementation evidence; EHR/data-infrastructure feasibility.
Smith et al., 2024 [61]	Netherlands	Pilot feasibility study	47 women enrolled; 39 completed the 4-week intervention.	Digital PCC; uptake	Among 39 completers, 16 (41%) attended a PCC consultation; usability and satisfaction were high.	Small uncontrolled pilot; original neighbourhood criterion was dropped because of recruitment difficulties; intended effectiveness study not yet performed.	Direct empirical PCC evidence; digital feasibility and acceptability only.
Dunlop et al., 2025 [62]	United States	Programme evaluation	Primary-care patients; *n* = 46	Virtual PCC risk assessment and counselling	The virtual programme achieved high engagement with digital risk assessment and counselling and increased participants’ confidence in fertility-related discussions.	Small uncontrolled programme; short-term engagement outcomes; no comparative clinical-effectiveness assessment.	Direct empirical PCC evidence; virtual PCC feasibility and engagement only.
Morita et al., 2026 [63]	Japan	Pilot feasibility study	Working women; *n* = 277	Digital PCC; occupational-health delivery	A PCC digital tool delivered through occupational health check-ups showed high user-reported usefulness and improved understanding of preconception health.	Satisfaction/knowledge outcomes; no long-term behavioural or clinical-effectiveness evidence.	Direct empirical PCC evidence; digital feasibility and acceptability only.
Fransen et al., 2018 [64]	Netherlands	Cross-sectional study	Women with low health literacy; *n* = 139 women; expert input used in study development	Health literacy; access; PCC counselling uptake	Most women (75%) were unaware of preconception counselling despite living in areas where invitations had been disseminated; nevertheless, attitudes towards participation were generally positive and 41% reported that they would participate. Intention was associated with perceived benefits, self-efficacy, subjective norms, and perceptions of existing knowledge and reproductive risk.	Selected population of women with low health literacy; cross-sectional design; self-reported determinants; single-country setting.	Direct empirical PCC evidence; awareness, health-literacy-related accessibility, and determinants of counselling participation.
Nypaver et al., 2016 [65]	United States	Narrative review	Women and families; N/A	Patient/population barriers; awareness	Advocates population-level awareness and community education to normalise preconception-health discussions and improve engagement.	Narrative synthesis; limited direct evaluation of proposed population strategies.	Narrative PCC synthesis; patient awareness and population-level implementation context.
Teshome et al., 2020 [66]	Ethiopia	Qualitative study	Women and healthcare providers; *n* = 13 key informant interviews	Barriers to PCC uptake	Low awareness, cultural norms and limited healthcare access were identified as major barriers to PCC uptake.	Small purposive sample; highly context-specific setting.	Direct empirical PCC evidence; LMIC patient/provider implementation barriers.
Munro et al., 2013 [67]	Canada	Qualitative exploratory study	Rural maternity-care professionals; 55 interview participants; 18 focus groups	Interprofessional collaboration; roles; funding	Collaboration between physicians, midwives and other maternity professionals was influenced by professional scope, funding arrangements and trust.	Maternity rather than PCC-specific setting; rural Canadian context.	Indirect empirical evidence from maternity care; used only to contextualise role allocation, professional trust and funding-related barriers.

DCE, discrete choice experiment; EHR, electronic health record; GP, general practitioner; LMIC, low- and middle-income country; OB-GYN, obstetrician–gynaecologist; PCC, preconception care; WTP, willingness to pay. The final structured evidence synthesis comprised 61 publications. Fifty-five publications directly addressed PCC or closely related preconception, interpregnancy, organisational, clinical, implementation, policy, access, equity, or economic dimensions, while six publications provided indirect contextual evidence from adjacent primary, reproductive, maternity, or prenatal healthcare settings. Indirect evidence was retained selectively when it informed predefined organisational or health-system domains, including interprofessional collaboration, financing, affordability, access, or implementation. Such evidence was used for contextual interpretation only and was not interpreted as evidence of PCC-specific clinical effectiveness. The classification in the “Role in the evidence synthesis” column reflects a structured qualitative assessment of evidence type and directness and does not constitute a formal quality or risk-of-bias rating.

**Table 2:** Evidence-informed comparative overview of PCC delivery models and their potential roles within integrated preconception care.

PCC Delivery Model	Evidence-Informed Contribution	Reported Strengths and Contextual Limitations	Authors’ Interpretative Synthesis: Potential Role Within PCC	Supporting References
**GP-led/primary care**	Population-level preventive care; early identification of modifiable preconception risk factors; chronic disease optimisation; medication review; continuity across the reproductive life course.	**Strengths:** high accessibility, continuity of care, holistic management, and established patient–provider relationships. **Limitations:** restricted consultation time, competing preventive priorities, insufficient PCC-specific training, and variable confidence in initiating reproductive-health discussions.	Potential first-contact role for universal or opportunistic PCC risk identification and initial management, including optimisation of chronic conditions, with referral when specialist reproductive, genetic, or medical assessment is required.	[13,22,27]
**OB-GYN-led/specialist care**	Specialist reproductive risk assessment and optimisation; management of complex reproductive and medical conditions; genetic counselling and carrier screening; preconception medication optimisation.	**Strengths:** specialised expertise in reproductive medicine, fertility, genetic risk, and management of complex or high-risk conditions. **Limitations:** accessibility outside pregnancy may be lower; services may be more strongly oriented toward women with established reproductive risk; specialist-led care may require greater healthcare resources and is less suited to population-wide PCC delivery.	Potential specialist role for women with increased reproductive, medical, genetic, or obstetric risk rather than a universally applicable entry point for all PCC.	[20,29,30,31,39]
**Midwife-/nurse-led care**	Person-centred preventive counselling; health promotion; reproductive-health education; identification of reproductive intentions and preconception-health needs.	**Strengths:** holistic and person-centred care, strong communication, continuity, health education, and preventive counselling. **Limitations:** professional scope, autonomy, regulation, education requirements, and funding vary substantially across healthcare systems, which may limit transferability of midwife-/nurse-led models between settings.	Potential role in accessible community-based PCC, health promotion, counselling, and continuity of care where professional regulation, training, and healthcare-system organisation support this scope of practice.	[11,17,21,34,40]
**GP–midwife collaborative model**	Integration of primary-care accessibility and continuity with reproductive-health counselling, health promotion, and complementary professional expertise.	**Strengths:** may combine accessibility with specialised reproductive counselling, facilitate coordinated referral, and strengthen continuity and comprehensiveness of preventive care. **Limitations:** implementation depends on clear professional roles, effective interprofessional communication, structured referral pathways, and organisational support; direct comparative evidence on model effectiveness remains limited.	Potential integrated primary-care model in systems where GPs and midwives have established complementary roles, appropriate scope of practice, and shared referral pathways.	[13,21,34,35]
**OB-GYN–midwife collaborative model**	Combination of specialist reproductive expertise with person-centred counselling, continuity of care, and coordinated multidisciplinary management.	**Strengths:** may combine specialist assessment with continuity and coordinated reproductive and genetic counselling, particularly for women with more complex needs. **Limitations:** broader implementation may be constrained by specialist availability, dependence on referral pathways, higher resource requirements, and costs; evidence specifically evaluating this configuration in PCC remains limited.	Potential targeted model for women with increased reproductive, medical, or genetic risk, particularly within specialist or referral-based services rather than as a universal population-level PCC model.	[29,30,31,36,39]

GP, general practitioner; OB-GYN, obstetrician–gynaecologist; PCC, preconception care. Evidence-informed contributions, strengths, and contextual limitations were synthesised from the cited literature. The proposed potential roles of the different provider models represent the authors’ interpretative synthesis of this evidence and should not be considered an externally or empirically validated organisational framework.

**Table 3:** Comparative synthesis of factors influencing women’s preferences for preconception care providers across healthcare contexts.

Preference Factor	Evidence Base	Variation Across Healthcare Systems/Study Contexts	Consistency and Directness of Evidence	Evidence-Informed Interpretation	Supporting References
**Professional expertise**	Surveys, qualitative studies, and discrete choice experiments	Preference for a specific professional group varies by setting. OB-GYNs were frequently preferred where reproductive specialist expertise was highly valued, whereas acceptance of GPs and midwives varied according to healthcare-system organisation, previous care experiences, accessibility, and continuity.	Relatively consistent evidence for the importance of perceived expertise; provider-specific preferences are context-dependent.	Women appear to value recognised reproductive-health competence more consistently than any single professional category. Provider preference should therefore not be interpreted as universally favouring one profession.	[6,32,46]
**Accessibility**	Qualitative studies, surveys, and discrete choice experiments	The importance of accessibility is shaped by how PCC is organised within routine healthcare, including appointment availability, opportunities for opportunistic counselling, and whether PCC is embedded within primary or reproductive healthcare services.	Relatively consistent evidence across different study designs, although operational definitions of accessibility vary.	Convenient and timely access appears to facilitate PCC uptake regardless of provider type. Integration within existing healthcare contacts may be more transferable across systems than creation of separate PCC services.	[32,42,46]
**Trust and perceived competence**	Qualitative studies and surveys	Although the professional group considered most trustworthy may differ between settings, women repeatedly value confidence in the provider, reliable information, respectful communication, and personalised counselling.	Consistently reported across multiple studies; predominantly observational and qualitative evidence.	Trust and perceived competence appear to be more stable determinants of provider preference than professional title alone.	[41,42,43,44]
**Affordability and financial incentives**	Discrete choice experiment and survey-based preference evidence	The effect of cost is likely to depend strongly on insurance coverage, availability of publicly funded services, household resources, and the extent of out-of-pocket payment within the healthcare system.	Limited and context-dependent evidence; largely based on stated preferences rather than observed utilisation.	Lower patient costs and financial protection may increase acceptability of PCC, but the magnitude of this effect cannot be generalised across financing systems.	[32]
**Confidentiality and privacy**	Qualitative studies	The organisational mechanisms ensuring confidentiality differ between settings, but concerns regarding privacy, non-judgemental communication, and sensitive discussion of reproductive intentions recur across studies.	Relatively consistent qualitative evidence; limited comparative evidence between healthcare systems.	Confidentiality appears to be a broadly relevant condition for acceptable PCC communication, although implementation is context-specific.	[42,43]
**Continuity of care**	Qualitative studies and surveys	Continuity is particularly relevant in systems where patients have established longitudinal relationships with GPs, midwives, or other reproductive-health providers; its importance may be less directly transferable to fragmented or episodic care settings.	Moderately consistent observational and qualitative evidence; direct comparative evidence is limited.	Established patient–provider relationships may facilitate discussion of reproductive intentions and sustained engagement, but the feasibility of continuity depends on healthcare-system organisation.	[41,42,43,44]

DCE, discrete choice experiment; GP, general practitioner; OB-GYN, obstetrician–gynaecologist; PCC, preconception care. The categories describing consistency and directness of evidence represent a structured qualitative interpretation of the included literature and are not formal quality or risk-of-bias ratings. Differences between healthcare systems are reported where supported by the available evidence; absence of a reported difference should not be interpreted as evidence of equivalence between settings.

**Table 4:** Evidence-informed synthesis of barriers and potential facilitators affecting the implementation of preconception care.

Level of Implementation	Key Barriers Reported in the Literature	Potential or Commonly Proposed Facilitators	Consistency and Context of Evidence	Supporting References
**Individual/provider level**	Limited knowledge of PCC guidelines; insufficient undergraduate or postgraduate training; low confidence in initiating reproductive-health discussions; reliance on individual clinical experience rather than standardised PCC protocols.	PCC-specific undergraduate and postgraduate education; continuing professional development; structured communication and risk-assessment tools; mentoring and professional support.	Consistently reported across multiple provider-focused studies and settings. Knowledge, training, and confidence barriers recur across the literature, although their magnitude varies by profession and healthcare context. Evidence supporting educational interventions as facilitators is plausible and implementation-oriented, but comparative evidence regarding the most effective training strategy remains limited.	[21,22,23,58]
**Organisational level**	Limited consultation time; high workload; absence of dedicated PCC consultations; fragmented multidisciplinary collaboration; unclear allocation of responsibilities between GPs, midwives, OB-GYNs, and other professionals.	Standardised clinical pathways; dedicated or opportunistic PCC encounters; clearly defined professional responsibilities; structured referral pathways; multidisciplinary collaboration.	Consistently reported but strongly influenced by local service organisation. Time constraints, competing priorities, and unclear professional roles recur across several settings. Proposed organisational facilitators are widely supported conceptually, although specific multidisciplinary configurations have rarely been compared directly.	[18,23,27,28,58,59]
**Health-system level**	Lack of dedicated reimbursement mechanisms or billing codes; limited public funding; absence or inconsistent implementation of national PCC guidance; fragmented integration into primary care; unequal distribution of preventive healthcare resources.	Public or insurance-based reimbursement; dedicated financing mechanisms; national PCC guidance and implementation standards; integration into routine preventive and reproductive healthcare; equity-oriented allocation of resources.	Recurrently reported but highly healthcare-system dependent. Financing and policy barriers are particularly sensitive to insurance coverage, primary-care organisation, national regulation, and resource availability. Evidence supports their importance as structural determinants, but there is insufficient comparative evidence to identify a universally superior financing or policy model.	[3,47,48,57,58]
**Digital/technological level**	Variable digital literacy; unequal access to digital technologies; limited integration of digital tools into routine clinical workflows; insufficient interoperability; data privacy and information-governance concerns.	EHR-based prompts and reminders; telehealth; mobile health applications; digital risk assessment; personalised digital counselling and decision support.	Emerging and context-dependent evidence. Current studies predominantly address feasibility, acceptability, engagement, and implementation rather than sustained clinical effectiveness. Digital facilitators should therefore be considered complementary rather than established substitutes for conventional PCC delivery.	[11,17,60,61,62,63]
**Patient/population level**	Limited awareness of PCC; low health literacy; unplanned pregnancy; financial barriers; cultural and socioeconomic constraints; limited perceived need for care before conception.	Population-level awareness campaigns; community outreach; school- and community-based health education; appropriately designed social-media interventions; normalisation of discussions about reproductive intentions and preconception health.	Several barriers are consistently reported, whereas their relative importance varies across populations. Limited awareness and health literacy recur across different settings, while financial, cultural, and socioeconomic barriers are more strongly context-dependent. Evidence for specific population-level facilitators remains heterogeneous and is less robust than evidence documenting the barriers themselves.	[41,46,64,65,66]

CPD, continuing professional development; EHR, electronic health record; GP, general practitioner; OB-GYN, obstetrician–gynaecologist; PCC, preconception care. The categories describing consistency and context of evidence represent a structured qualitative synthesis and are not formal quality or risk-of-bias ratings. “Potential or commonly proposed facilitators” includes strategies identified in empirical studies, reviews, guidelines, or implementation literature; their inclusion should not be interpreted as evidence that all strategies have demonstrated comparative effectiveness.

## Data Availability

No new datasets were generated or analysed during the current study. This narrative review is based on previously published studies, guidelines, reports, and publicly available sources, all of which are cited in the reference list. Data sharing is therefore not applicable to this article.

## Supplementary Materials

The following supporting information can be downloaded at: https://www.mdpi.com/article/10.3390/healthcare14172706/s1, Table S1: Reconstructed database-specific search strategies and documented search outputs for PubMed, Scopus, and Web of Science Core Collection.

## Abbreviations

CPD	Continuing Professional Development
DCE	Discrete Choice Experiment
EHR	Electronic Health Record
GP	General Practitioner
LMIC	Low- and Middle-Income Country
OB-GYN	Obstetrician–Gynaecologist
PCC	Preconception Care
WTP	Willingness to Pay
