# Supplementary material for: Organisation, Financing, and Implementation of Preconception Care in Primary Care: Implications for Women’s Health and Well-Being
Source: Healthcare (Basel). 2026 Aug 25;14(17):2706. doi: 10.3390/healthcare14172706 (PMC13565262; doi:10.3390/healthcare14172706)
Supplement: Supplementary file 1 [file healthcare-14-02706-s001.zip › healthcare-4467908-supplementary.pdf]

Table S1. Reconstructed database-specific search strategies and documented search outputs for PubMed, Scopus, and Web of Science Core Collection.

**Database**	**Search strategy**	**Records identified**
**PubMed**	*("Preconception Care"[MeSH Terms] OR "preconception care"[tiab] OR "preconception counselling"[tiab] OR "preconception counseling"[tiab] OR "preconception health"[tiab]) AND (provider[tiab] OR "general practitioner*"[tiab] OR obstetrician*[tiab] OR gynaecologist*[tiab] OR gynecologist*[tiab] OR midwi*[tiab] OR nurse*[tiab] OR "primary care"[tiab] OR "multidisciplinary care"[tiab] OR "midwife-led care"[tiab] OR "nurse-led care"[tiab] OR "provider preference*"[tiab] OR "preferred provider*"[tiab] OR "trusted provider*"[tiab] OR "healthcare provider choice"[tiab] OR financ*[tiab] OR reimbursement[tiab] OR affordability[tiab] OR "willingness to pay"[tiab] OR "contingent valuation"[tiab] OR "discrete choice experiment*"[tiab] OR "implementation barrier*"[tiab] OR "primary care barrier*"[tiab] OR "GP perspective*"[tiab] OR "GP readiness"[tiab] OR "GP confidence"[tiab] OR facilitator*[tiab] OR "digital health"[tiab] OR telehealth[tiab])	**320**
**Scopus**	*TITLE-ABS-KEY ("preconception care" OR "preconception counselling" OR "preconception counseling" OR "preconception health") AND TITLE-ABS-KEY (provider OR "general practitioner*" OR obstetrician* OR gynaecologist* OR gynecologist* OR midwi* OR nurse* OR "primary care" OR "multidisciplinary care" OR "midwife-led care" OR "nurse-led care" OR "provider preference*" OR "preferred provider*" OR "trusted provider*" OR "healthcare provider choice" OR financ* OR reimbursement OR affordability OR "willingness to pay" OR "contingent valuation" OR "discrete choice experiment*" OR "implementation barrier*" OR "primary care barrier*" OR "GP perspective*" OR "GP readiness" OR "GP confidence" OR facilitator* OR "digital health" OR telehealth)**	**280**
**Web of Science Core Collection**	*TS=("preconception care" OR "preconception counselling" OR "preconception counseling" OR "preconception health") AND TS=(provider OR "general practitioner*" OR obstetrician* OR gynaecologist* OR gynecologist* OR midwi* OR nurse* OR "primary care" OR "multidisciplinary care" OR "midwife-led care" OR "nurse-led care" OR "provider preference*"	**205**

	OR "preferred provider*" OR "trusted provider*" OR "healthcare provider choice" OR financ* OR reimbursement OR affordability OR "willingness to pay" OR "contingent valuation" OR "discrete choice experiment*" OR "implementation barrier*" OR "primary care barrier*" OR "GP perspective*" OR "GP readiness" OR "GP confidence" OR facilitator* OR "digital health" OR telehealth)	
**Language**	English	—
**Search period**	January 2010–March 2026; final search update conducted on 14 July 2026	—
**Publication types considered**	Original research studies, systematic reviews, scoping reviews, narrative reviews, clinical guidelines, policy reports, and relevant methodological or health-economic publications	—
**Additional identification methods**	Reference-list screening and manual searches	**27**
**Total records identified through database searching**	PubMed + Scopus + Web of Science	**805**
**Total records identified before duplicate removal**	Database records plus records identified through reference-list screening and manual searches	**832**

*The original saved database search histories were not retained. The database-specific formulations presented in this table were reconstructed during manuscript revision from documented search terms and Boolean combinations used during the original search process and were adapted to the syntax of each database. They should therefore be interpreted as reconstructed representations of the original searches rather than as verbatim exports of the original database search histories. The reported record counts correspond to the final documented database outputs following the July 2026 search update. Because database content and indexing change over time, rerunning the reconstructed formulations may not reproduce the historical record counts exactly.
